# A multimodal HPLC stability indicating approach for the estimation of Semaglutide and Tirzepatide in bulk, pharmaceutical dosage forms, and rat plasma: a six-edged sustainability appraisal

**DOI:** 10.1186/s13065-025-01716-7

**Published:** 2026-01-27

**Authors:** Hadeel A. Khalil, Nermeen A. Hassanein, Amira F. El-Yazbi, Hoda Mahgoub

**Affiliations:** Pharmaceutical Analytical Chemistry Department, Faculty of Pharmacy, Alexandria University, 1 El Khartoum Square, Alexandria, 21521 Egypt

**Keywords:** Semaglutide, Tirzepatide, HPLC, Stability indicating, Plasma, Sustainable analysis

## Abstract

Over the recent years, there has been a notable surge in consumer demand for rapid and effective weight-loss pharmaceuticals that are also capable of managing type 2 diabetes. Owing to their exceptional efficacy, GLP-1 receptor agonists, including Tirzepatide (TIR) and Semaglutide (SEM), have had phenomenal outcomes and confidence among consumers. A rapid, straightforward, and thorough approach for quantifying SEM and TIR is essential for quality control purposes given the rising use of these drugs in the pharmaceutical industry. This work presents the first stability-indicating HPLC method for quantifying TIR and SEM under various stress conditions (acidic and basic hydrolysis, oxidative, and photolytic degradation) without interference from degradants. In addition, the proposed methods are capable of accurately quantifying each drug in bulk, pharmaceutical dosage forms, and spiked plasma. The analysis of TIR and SEM was performed using an Inertsil ODS-3 (4.6 × 250 mm, 5 μm particle size) C18 column and the elution of the drugs was achieved isocratically using 0.1% formic acid (pH 2.5) and ACN in the ratio 30:70 with a flow rate 1 mL/min using DAD detector at 220 nm with SEM and TIR eluting at 1.42 and 1.68 min respectively. The proposed method was validated in line with the International Conference of Harmonization (ICH) guidelines and has demonstrated excellent accuracy, linearity, and superior sensitivity with LOD values of 10 and 16 ng/mL for TIR and for SEM, respectively. The obtained linearity range for both TIR and SEM was 1–500 µg/mL with correlation coefficients > 0.9999. An in-depth six- edged sustainability assessment of the proposed method was conducted using greenness, whiteness, blueness and violet innovation metrics was performed using Analytical Greenness Metric (AGREE), Modified Green Analytical Procedure Index (MoGAPI), Analytical Eco-scale, Analytical Green Star Area (AGSA), Carbon Footprint Reduction Index (CaFRI), Whiteness using RGB algorithm, Blue Applicability Grade Index (BAGI), Click Analytical Chemistry Index (CACI), Violet Innovation Grade Index (VIGI) tools and Stability Toolkit for the Appraisal of Bio/Pharmaceuticals’ Level of Endurance (STABLE).

**Supplementary Information:**

The online version contains supplementary material available at 10.1186/s13065-025-01716-7.

## Introduction

Obesity has been classified as a serious public health emergency as it currently stands as the fifth leading cause of death worldwide [1]. Obesity is one of the main lifestyle diseases that leads to a series of health issues that eventually result in several chronic diseases, ranging from diabetes, cardiovascular disease, and cancer [2]. In the recent years, the demand for weight reduction drugs that offer rapid and effective weight loss while effectively managing type 2 diabetes has shot up dramatically. Among all the weight loss medications available on the market, Glucagon-like peptide-1 (GLP-1) receptor agonists have been the most successful in gaining confidence among consumers [3–5].

GLP-1 receptor agonists are a novel class of antidiabetic agents that have demonstrated remarkable efficacy in treating type 2 diabetes by stimulating the secretion of insulin from beta cells and diminishing the glucagon secretion in hyperglycemic states. Besides, GLP-1 receptor agonists reduce appetite therefore it is an effective weight loss agents [6]. The most recent US Food and Drug Administration (FDA) – approved drugs in this class are Semaglutide (SEM) and Tirzepatide (TIR).

SEM can be administered either daily as oral tablets (Rybelsus^®^) or once weekly as injectable dosage forms (Ozempic^®^ and Wegovy^®^) [7–9]. Moreover, SEM has demonstrated effectiveness in decreasing systolic blood pressure [4] and providing a neuroprotective effects [10].

TIR is currently available as once weekly injections marketed under the brand name Mounjaro^®^. In contrast to SEM, Tirzepatide (TIR) exhibits a dual impact which provides superior weight reduction and glycemic management. By targeting key incretin pathways, TIR functions as a glucose-dependent insulinotropic polypeptide (GIP) and GLP-1 receptor agonist [11]. According to a recent comparative study on adults with obesity, TIR is superior on SEM in weight loss [12].

Reviewing the literature, it was found that only three methods have been described for TIR estimation namely a spectrofluorimetric method [13], and two high-performance liquid chromatographic method with diode array detection (HPLC-DAD). In the spectrofluorimetric method, TIR and SEM were quantified distinctly using ethanol as a solvent. On the other hand, the first method HPLC method was stability indicating method and the paper applied very harsh conditions with insignificant degradation results, and the percentage degradation was ranging from 0.73 to 8.73% [14] which does not comply with ICH guidelines [15] and the other developed HPLC method for TIR quantitation employed gradient mobile phase elution and a relatively long separation time (30 min) [16].

As for SEM, an HPLC-DAD was reported to investigate the degradation of SEM under various stress conditions [17]. However, the obtained percentage degradation of SEM using this method were insignificant as they were less than 10% which does not comply with the ICH guidelines(10–20%) [15]. Another HPLC-DAD method was described for SEM estimation in presence of pioglitazone and metformin [18]. Furthermore, an HPLC method with fluorescence detection [19], liquid chromatography with tandem mass detection (LC-MS/MS) [20], ultra-performance liquid chromatography (UPLC) [21] and spectrophotometric methods [22] were reported for SEM determination in bulk and pharmaceutical dosage forms.

**Fig. 1:**
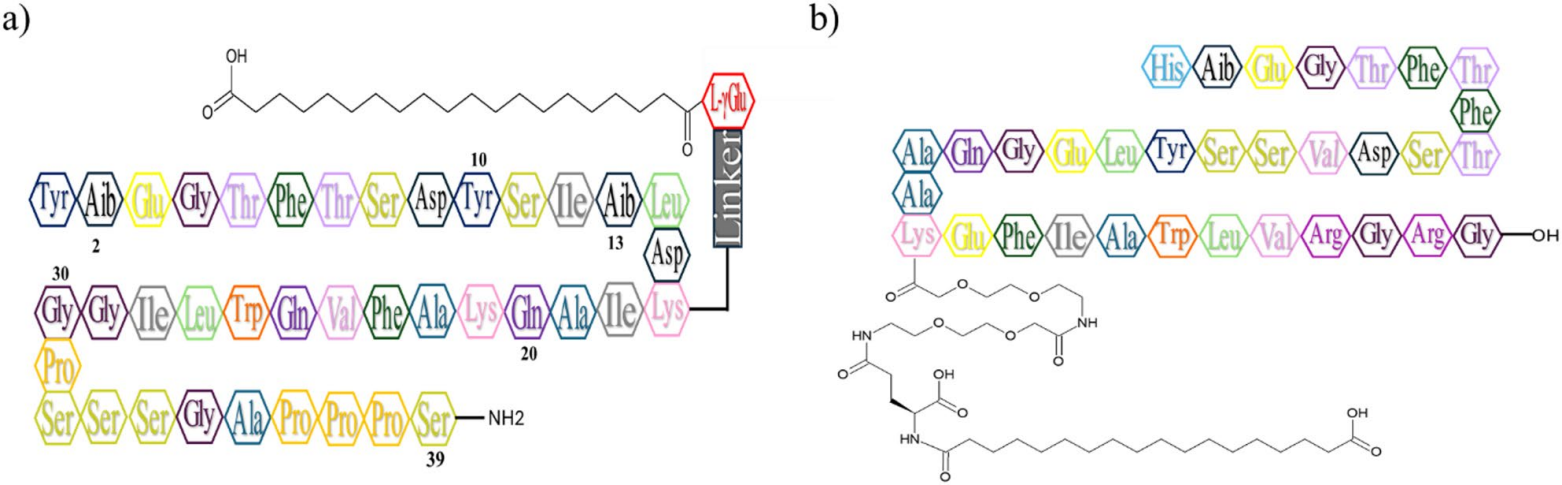
Chemical structure of **a** TIR and **b** SEM

Due to the rising consumption of pharmaceutical dosage forms containing either SEM (C_187_H_291_N_45_O_59_) or TIR (C_225_H_348_N_48_O_68_) (Fig. 1) by patients, the development of a fast, straightforward, and comprehensive method to study the quantification of TIR and SEM is crucial for quality control studies. To the best of our knowledge, no techniques in the literature were described for the quantification of TIR and SEM in presence of their degradation products under different stress conditions. This work aims to develop a robust stability indicating HPLC method capable of quantifying each drug under different stress conditions without interference with its degradants. Moreover, the proposed method was effectively employed to determine each drug in bulk, pharmaceutical dosage forms, and spiked rat plasma. Additionally, the proposed method was validated in accordance with the ICH guidelines [23]. Finally, an extensive hexagonal sustainability evaluation of the proposed method was performed by implementing several greenness, whiteness, blueness, innovation, carbon footprint and stability metrics including AGREE, MoGAPI, Analytical Eco-scale, AGSA, CaFRI, Whiteness using RGB algorithm, BAGI, CACI, VIGI tools and STABLE.

## Experimental

### Instrumentation

The HPLC-DAD system consisted of Agilent 1260 Infinity Series (auto-injector, quaternary pump, vacuum degasser and diode array and multiple wavelength detector G1315 C/D and G1365 C/D) connected to a computer loaded with Openlab CDS software (Agilent Technologies, Santa Clara, CA, USA).

### Materials

SEM was provided by Novo Nordisk pharmaceutical company (Cairo, Egypt), while TIR was provided by Eli Lilly company scientific office (Cairo, Egypt). Ozempic^®^ injection (1.0 mg of Semaglutide), and Mounjaro^®^ injection (10.0 mg of Tirzepatide) were purchased from the local market.

HPLC-grade formic acid and acetonitrile (ACN) were purchased from Thermo Fisher Scientific (USA). Analytical grade hydrochloric acid (HCl), sodium hydroxide (NaOH), and hydrogen peroxide(H_2_O_2_) were purchased from El Gomhorreya Chemicals Co, Egypt. High purity deionized water was used.

### Chromatographic conditions

The chromatographic separation was achieved using an Inertsil ODS-3 (4.6 × 250 mm, 5 μm particle size) column (GL Sciences, Japan). The mobile phase was composed of 0.1% Formic acid (pH 2.5) in water and ACN eluted isocratically in the ratio 30:70 at a flow rate of 1 mL/min. Five microliters of the sample were injected and monitored by a diode array detector from 220 nm to 300 nm. The DAD wavelength was set at 220 nm for SEM and TIR detection. All measurements were performed at 25 °C. All pH measurements were done using a Jenway 3510 pH meter. Calibration was performed using standard buffers of pH 4 and 7 at room temperature.

### Preparation of standard solutions

Separate stock solutions of 1000 µg/mL were prepared in deionized water for TIR and SEM. Aliquots of the stock solutions were further diluted with deionized water to obtain working solutions with a concentration range from 1 to 500 µg/mL for TIR and SEM, respectively. Each determination was performed three times as described in the aforementioned chromatographic conditions. The calibration graphs were constructed by plotting the peak areas against their corresponding concentrations of TIR and SEM and the regression equations were generated.

### Assay in pharmaceutical dosage forms

A single dose of Mounjaro^®^ injection (10.0 mg) and Ozempic^®^ injection (1.0 mg) was separately diluted in a 100 mL volumetric flask using deionized water, vortexed for 10 min, filtered, and diluted with deionized water to obtain the concentrations within the specified ranges. Samples were then injected under the aforementioned chromatographic conditions, and corresponding recoveries were calculated from the calibration graphs.

### Assay in plasma

Blank rat plasma was obtained from control rats used in a prior study that was conducted according to an experimental protocol approved by the Institutional Animal Care and Use Committee at Alexandria University (Ethical Approval No. AU/ 0620234121151) [24]. Animals were euthanized by exsanguination following deep anesthesia using 50 mg/Kg intraperitoneal injection of thiopental. Blood samples were collected during exsanguination and centrifuged at 5000 rpm for 10 min to separate the plasma. Collected plasma was stored at -80 °C.

100 µL rat plasma aliquots were transferred into a set of eppendorfs to which appropriate aliquots of TIR and SEM were added to produce solutions in the range of 1–500 µg/mL for TIR and SEM. This was followed by the addition of 300 µL of ACN as a precipitating agent. Each sample solution was vortexed for 2 min, centrifugated at 13,500 rpm for 5 min. The supernatant of each solution was transferred to clean test tubes, and evaporated till dryness. This was followed by reconstitution of the obtained residue with 100 µL of deionized water. The obtained solution was then vortexed, filtered, and then injected to the column under the above-mentioned conditions. Blank plasma experiments were carried out simultaneously. The calibration graphs were constructed by plotting the peak areas against their corresponding concentrations of TIR and SEM in plasma.

### Forced degradation studies

Forced degradation studies of TIR and SEM were conducted under different conditions, including hydrolytic (acid, alkaline), oxidation, and photolytic conditions in accordance with the ICH guidelines [15].

#### Acidic degradation

In separate volumetric flasks, aliquots of TIR and SEM stock solutions were mixed with 1mL of 0.1 M HCl at room temperature and kept for 10 min. The solutions were then neutralized to pH 7 using an alkali and diluted with deionized water to obtain a concentration of 100 µg/mL.

#### Basic degradation

In separate volumetric flasks, aliquots of TIR and SEM stock solutions were mixed with 1mL of 0.1 M NaOH. SEM-containing solutions were left at room temperature for 5 min, whereas TIR solutions were left for 20 min at room temperature. The solutions were then neutralized to pH 7 using an acid and diluted with deionized water to obtain a concentration of 100 µg/mL.

#### Wet heat degradation

Under reflux at 60 °C, aliquots of TIR and SEM stock solutions were mixed with 1 mL of deionized water and left for 60 and 90 min, respectively. The solutions were then diluted with deionized water to obtain a concentration of 100 µg/mL.

#### Oxidative degradation

In separate volumetric flasks, aliquots of TIR and SEM stock solutions were treated with 1 mL of 10% H_2_O_2_ at room temperature. SEM-containing solutions were left for 20 min while TIR-containing solutions were left for 60 min. The solutions were then diluted with deionized water to obtain 100 µg/mL of both TIR and SEM.

#### Photolytic degradation

Aliquots of TIR and SEM stock solutions were subjected to direct sunlight for 24 h and were then diluted with deionized water to obtain a concentration of 100 µg/mL.

All samples were then injected to the HPLC column under the optimized chromatographic conditions and from the generated peak areas of TIR and SEM in each analyzed sample, the percentage of degradation was calculated.

## Results and discussion

Stability-indicating assays frequently deploy HPLC owing to its effectiveness in separating numerous components with high sensitivity, specificity, and resolution. The development of a stability-indicating assay method (SIAM) as described by the ICH guidelines necessitates performing forced degradation of the studied drugs under different conditions including oxidation, pH, heat, light, and others, and the separation of the studied drug from its degradants is obligatory. Moreover, the degree of degradation of the drug under investigation should range from 10 to 20% [15].

This work attempts to develop and validate a new stability-indicating HPLC method for the determination of TIR and SEM in their raw material, pharmaceutical dosage forms, plasma as well as in presence of their potential degradation products under different stress conditions. Given the uncertain nature of the degradation products, forced degradation studies were taken into consideration in the chromatographic method development phase.

### Method development

The development of a multimodal HPLC method capable of quantifying TIR and SEM in different matrices as well as in the presence of their degradants avoiding any interference or overlapping necessitates the optimization of the chromatographic conditions and parameters to ensure the reliability of the obtained results.

#### Choice of the mobile phase

Initially, a mobile phase consisting of 0.1% formic acid in water and methanol was used in different ratios. However, broad peaks were obtained and consequently methanol was replaced by ACN. Increasing the proportion of ACN was found to be essential for eluting the selected drugs in an acceptable retention time and peak shape. None the less, further increase of ACN proportion greater than 70%, v/v leads to rapid elution of the selected drugs with poor peak symmetry and inadequate capacity factors. Eventually, it was concluded that isocratic elution of 0.1% formic acid (pH 2.5) and ACN in the ratio 30:70 resulted in the best peak shapes in reasonable retention times of 1.68 and 1.42 for TIR and SEM, respectively and hence was selected.

The proposed method is based on isocratic elution mode which owns several advantages including operation simplicity due to the constant composition of the mobile phase which is more convenient for UV detection with stable baseline. In addition, this elution mode provides better reproducibility and shorter analysis time compared to gradient elution mode [25].

#### Choice of the stationary phase

Multiple columns have been tried to choose the most convenient stationary phase that provides the best results. Owing to its less hydrophobic nature, a Fortis C8 column resulted in very short analysis (< 1 min) and hence was excluded. Moreover, columns with small particle size (3 μm) including the InertSustain C18 HPLC Column, (3 μm, 150 × 4.6 mm) have been tried, yet poor peak shapes in long run times were obtained. On the other hand, a notable improvement in the chromatographic separation was achieved using an Inertsil ODS-3 (4.6 × 250 mm, 5 μm particle size) C18 column and hence was selected as the optimum column.

#### Choice of flow rate

The flow rate effect was studied by trying various flow rates varying from 0.5 mL/min to 1.5 mL/min, and 1 mL/min was optimum, considering analysis time, peak shape, and the applied pressure of the column.

#### Choice of appropriate wavelength

TIR and SEM can be detected at several wavelengths in the selected mobile phase; however, for both drugs, the highest absorbance and, thus, the highest sensitivity was noted at 220 nm. Quantification was achieved using DAD based on the peak area measurement.

**Fig. 2:**
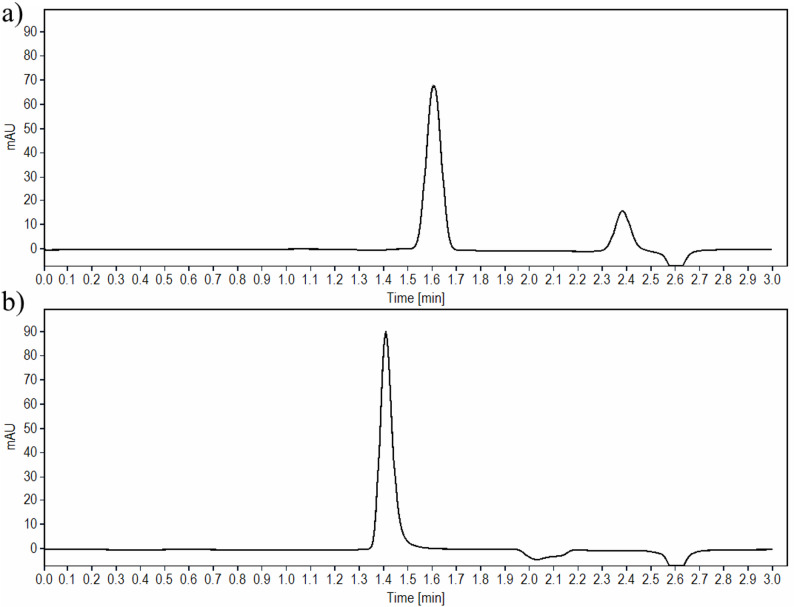
HPLC chromatograms of 5 µL injection of **a** 100 µg/mL TIR **b** 100 µg/mL SEM at 220 nm

Additionally, TIR and SEM and their respective stress degradation products were well separated by the previously indicated chromatographic conditions. Computation of the resolution and other system suitability parameters were determined to be satisfactory as listed in (Table 1). The obtained chromatograms upon analysis of TIR and SEM in their authentic standards using the proposed method are illustrated in Fig. 2.

**Table 1:** System suitability parameters for the proposed HPLC-DAD method

Parameter	TIR	SEM
t_R_	1.68	1.42
Capacity factor (K`)	3.00	2.50
Tailing Factor (T)	1.10	1.07
Theoretical plates (N)	3436	2071

^*^Recommendations by ICH guidelines: K`= 2–10, *N* > 2000, and T = 0.9–1.2 [23]

### Stability-indicating aspects

Several forced degradation experiments were conducted to investigate the degradation products of TIR and SEM and their chromatographic behavior. Hydrolysis in both acidic and basic conditions, wet heat, oxidative, and photolytic degradation studies were performed; each of them was conducted under specific conditions as shown in the Table 2.

**Table 2:** Summary of the forced degradation studies conducted on TIR and SEM and analyzed using the proposed HPLC-DAD method

Degradation Type	TIR					SEM				
	Degradation conditions	Duration	% Degradation	^a^t_*R*_ (mins)	^b^Rs	Degradation conditions	Duration	% Degradation	^a^t_*R*_ (mins)	^b^Rs
Acidic degradation	0.1 M HCl, 25 °C	10 min	16.90%	3.30	6.74	0.1 M HCl, 25 °C	10 min	12.00%	2.80	3.217
Basic degradation	0.1 M NaOH, 25 °C	20 min	18.97%	2.40, 3.30	7.58	0.1 M NaOH, 25 °C	5 min	13.00%	2.90, 3.05	4.09
Oxidative degradation	10% H_2_O_2_, 25 °C	60 min	15.91%	-	6.05	10% H_2_O_2_, 25 °C	20 min	14.80%	-	7.27
Photo-degradation	Direct light, 25 °C	24 h	11.84%	-	-	Direct light, 25 °C	24 h	12.35%	-	-
Thermal degradation (Wet heat)	Reflux, 60 °C	90 min	17.90%	-	-	Reflux, 60 °C	60 min	12.60%	-	-

^a^Retention times of degradation products

^b^Selectivity was calculated between the parent peaks and the most adjacent degradation product peak

**Fig. 3:**
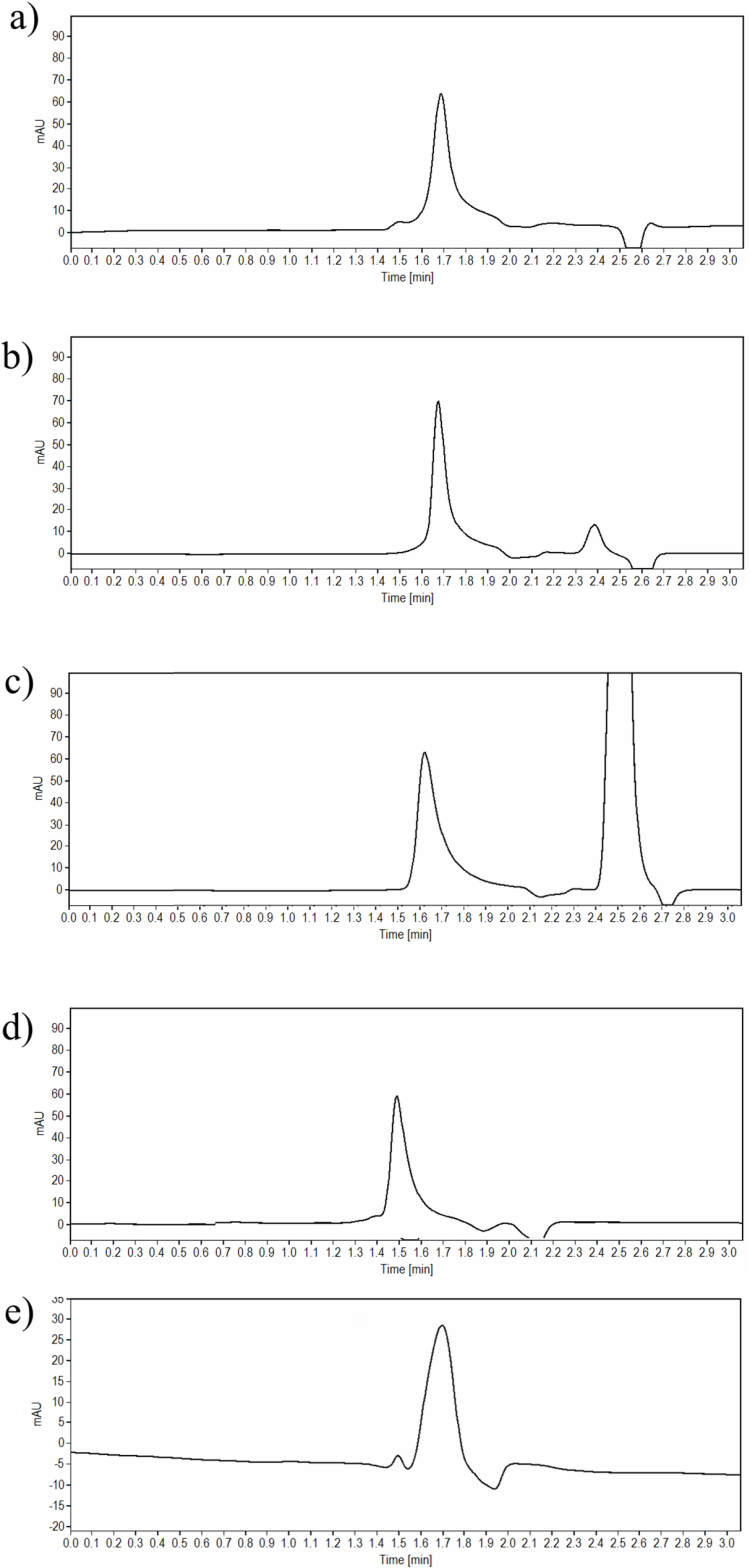
**a**-**e** HPLC chromatograms of 100 µg/ml solution of TIR after exposure to **a** acid degradation, **b** base degradation, **c** oxidative degradation, **d** photolytic degradation, and **e** thermal wet heat degradation

**Fig. 4:**
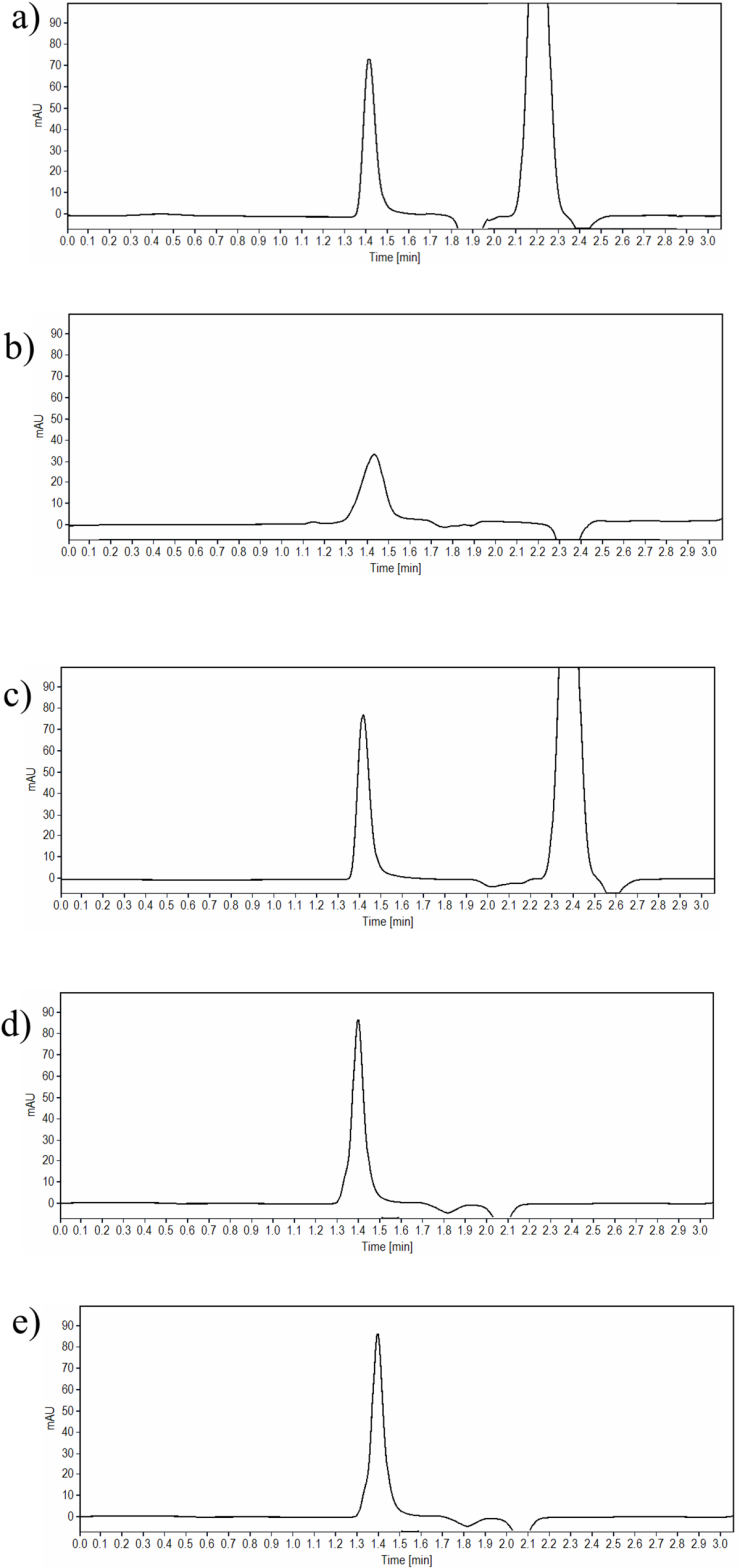
**a**-**e** HPLC chromatograms of 100 µg/mL solution of SEM after exposure to **a** acid degradation, **b** base degradation, **c** oxidative degradation, **d** photolytic degradation, and **e** thermal wet heat degradation

Exposure of the investigated drugs to strong acidic and basic media (1 M HCl and 1 M NaOH), high temperatures, and longer durations, has led to precipitation more likely due to denaturation of the peptides, and thus conducting the experiments at less harsh conditions was chosen. In contrast, reducing the concentration of the reagent to 0.1 M HCl or 0.1 M NaOH contribute to an adequate degradation of TIR and SEM. Keeping the solutions for 10 min in acidic conditions at room temperature, resulted in a percentage degradation of 16.90% and 12.00% for TIR and SEM, respectively. In addition, one degradation peak was observed was at 3.30 and 3.00 min for TIR and SEM, respectively (Fig. 3a for TIR and Fig. 4a for SEM).

In 0.1 M NaOH, the test solution was degraded within 20 min for TIR and within 5 min for SEM at room temperature and the percentage degradation was 18.97% and 13.00% for TIR and SEM, respectively. Of note, two extra degradation peaks have appeared at 2.40 min and 3.30 min for TIR, while for SEM, the two degradation peaks were observed at 2.50 and 3.05 min. The proposed chromatographic system was able to adequately separate both degradant peaks without any interference with the parent drug peak (Fig. 3b for TIR and Fig. 4b for SEM).

For oxidative degradation studies, the application of 10% H_2_O_2_ was selected as the addition of high concentration of H_2_O_2_ (30%) caused the precipitation of the drug solution. Exposure of the test solutions to 10% H_2_O_2_ led to TIR degradation of TIR by up to 15.91% when used at room temperature for one hour, and the characteristic peak of H_2_O_2_ was well-resolved from the drug peak (Rs value was 6.05) well-resolved from the parent drug peak. Similarly, exposure of SEM test solution to 10% H_2_O_2_ at room temperature for 20 min, led to a degradation up to 14.80% (Rs value was 7.27)(Fig. 3c for TIR and Fig. 4c for SEM).

Photolytic degradation was studied by exposure of the test solutions to direct light for 24 h, the obtained degradation was 11.84% and 12.35% for TIR and SEM, respectively, with no additional degradant peaks appearing in the chromatograms (Fig. 3d for TIR and Fig. 4d for SEM.

For wet heat degradation, the drug solutions were refluxed at 60 °C for 1.5 h and 1 h for TIR and SEM, respectively to investigate the impact of heat on the drug. The obtained reduction in the drug peak area was 17.90% and 12.60% for TIR and SEM, respectively when compared to a standard solution of the same concentration (Fig. 3e for TIR and Fig. 4e for SEM).

Resolution between any of the parent peaks and the most adjacent degradation product peak was computed in each of these experiments. The obtained resolution values did not fall below 2 suggesting that the parent drugs` peaks and the peaks of their degradation products have a sufficient baseline separation. It is important to note that DAD peak purity test findings verify that the TIR and SEM peaks are homogenous and pure in each of the analyzed samples that were examined under forced degradation conditions.

### Validation of the proposed method

The developed methods were validated in accordance with the ICH guidelines [23].

#### Linearity and concentration ranges

The linearity of the proposed method was investigated by analyzing different concentrations of TIR and SEM. The calibration graphs were subjected to the least square method to generate the linear regression equations. The measured peak areas were found to be proportional to the concentration of TIR and SEM under the studied chromatographic conditions. The performance data and statistical parameters, including linear regression equations, concentration ranges, correlation coefficients, standard deviations of the intercept (Sa), slope (Sb), and standard deviations of residuals (Sy/x) are displayed in Table 3. The high correlation coefficient values (> 0.9998), F values (41775 and 4654 for TIR and SEM, respectively) and low deviations around the slope (Sb% < 0.053%) confirms the good linearity of the developed methods.

**Table 3:** Analytical parameters for the determination of TIR and SEM using the proposed HPLC-DAD method

Parameter	TIR	SEM
Wavelength (nm)	220	220
Concentration range (µg/mL)	1-500	1-500
a	-2.05	-28.90
^a^S_a_	14.00	17.78
b	11.32	3.61
^b^S_b_	0.06	0.05
RSD% of the slope (*S*_*b*_%)	0.49	1.47
r	0.9999	0.9998
^c^S_y/x_	23.97	18.45
F	41,775	4654
^d^Significance F	3.4 × 10^− 9^	2.1 × 10^− 4^
^e^LOD (µg/mL)	0.016	0.010
^f^LOQ (µg/mL)	0.051	0.030

^a^Standard deviation of the intercept

^b^Standard deviation of the slope

^c^Standard deviation of residuals

^d^Variance ratio, equals the mean of squares due to regression divided by the mean of squares about regression (due to residuals)

^e^Limit of detection

^f^Limit of quantification

#### Detection and quantification limits

The limit of detection (LOD) is defined as the concentration that has a signal-to-noise ratio of 3:1, while the limit of quantification (LOQ) is the concentration at which the signal-to-noise ratio is 10:1. Excellent sensitivity of the developed methods is evident by the low LOD and LOQ values calculated for TIR and SEM (Table 3).

#### Accuracy and precision

Investigation of the intraday precision and accuracy were performed by analyzing nine samples at three different concentration levels of each drug in triplicates in the same day. Likewise, the interday precision and accuracy were examined however the analyses were performed on three separate days. The recoveries were calculated from the obtained regression equations and displayed in (Table 4). The percentage relative standard deviation (RSD%) and percentage relative error (Er%) did not exceed 2% confirming the high repeatability and accuracy of the proposed methods.

**Table 4:** Intra-day and inter-day accuracy and precision for the determination of TIR and SEM using the proposed HPLC-DAD method

Parameters		Tirzepatide (TIR)			Semaglutide (SEM)		
	Labeled concentration (µg/mL)	25	200	300	50	200	400
Intra-day	Found (µg/mL)	25.02	201.52	300.51	49.16	201.44	400.36
		24.39	201.65	300.78	49.16	200.60	399.53
		24.64	202.20	301.86	50.27	199.767	400.92
	Average (µg/mL)	24.68	201.79	301.05	49.53	200.60	400.27
	±SD^a^	0.31	0.36	0.72	0.64	0.83	0.70
	%Er^b^	-1.27	0.90	0.35	98.11	0.30	33.42
	%Recovery^c^	98.73	100.90	100.35	99.06	100.30	100.07
	%CV^d^	1.27	0.18	0.24	1.29	0.42	0.18

	Concentration (µg/mL)	25	200	300	50	200	400
Inter-day	Found (µg/mL)	24.68	200.75	301.05	49.53	200.60	400.27
		25.47	197.77	297.86	49.50	196.21	400.50
		24.56	200.54	299.67	48.36	199.49	399.98
	Average (µg/mL)	24.90	199.69	299.53	49.13	198.76	400.24
	±SD^a^	0.49	1.66	1.60	0.66	2.29	0.26
	%Er^b^	-0.38	-0.16	-0.16	-1.74	-0.62	0.06
	%Recovery^c^	99.62	99.84	99.84	98.26	99.38	100.06
	%CV^d^	1.98	0.83	0.53	1.35	1.15	0.07

^a^Standard deviation (*n* = 9)

^b^Percentage relative error

^c^Mean percentage recovery

^d^Percentage coefficient of variation

#### Selectivity and specificity

**Fig. 5:**
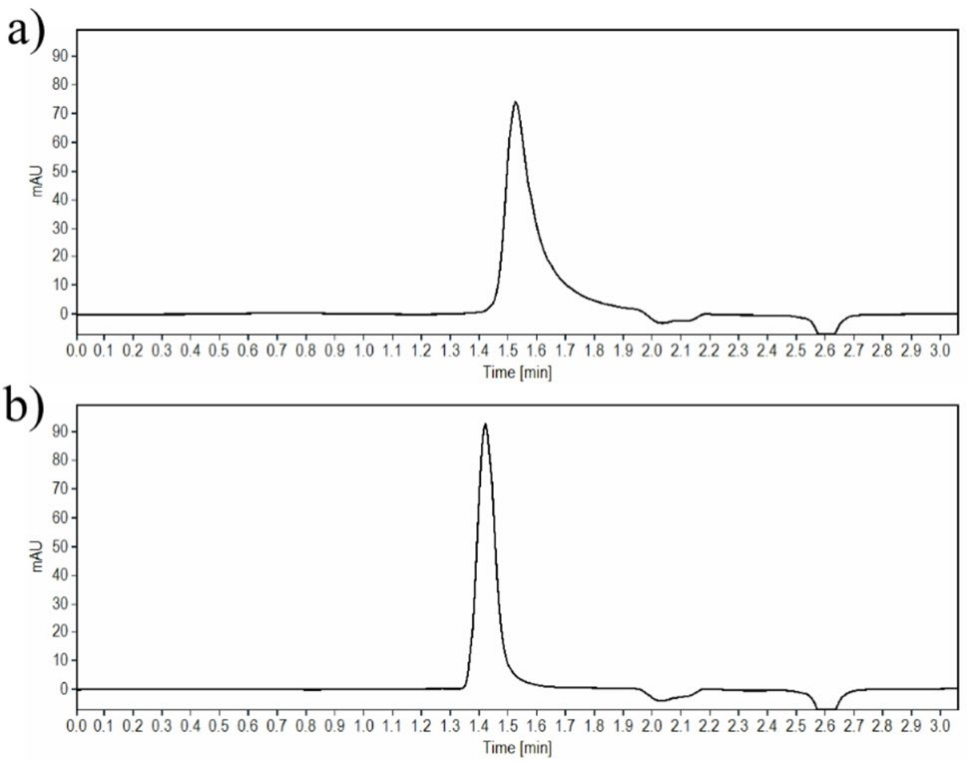
Chromatograms of TIR and SEM prepared from their dosage forms; **a** 100 µg/mL Mounjaro^®^ and **b** 100 µg/mL Ozempic^®^ respectively, measured at 220 nm

In order to assess the specificity of the developed methods; the chromatograms of the standard and injection dosage form samples were compared. As illustrated in Fig. 5; both the standard and injection test solutions have exhibited identical retention times for TIR and SEM with no interfering peaks from the excipients in the pharmaceutical dosage forms.

Additionally, the specificity of the developed methods is verified by the resolution of the intact drugs from their forced degradation products as shown in the chromatograms of the two drugs following different stress conditions (Figs. 3 and 4).

DAD, which uses the spectra of TIR and SEM standards as a reference to verify the purity of the peaks, was employed to validate the selectivity of the suggested methods. These methods showed to be selective in separating TIR and SEM from their forced degradation products considering that the computed purity angles did not surpass the threshold limit (Figure S1).

#### Robustness

Robustness was evaluated by testing the impact of small variations in different experimental conditions as mobile phase compositions (± 2%), flow rate (± 0.2 mL/min) and working wavelength (± 2 nm). No significant variations were observed on the responses nor the peaks` resolution. The obtained RSD% for the measured peak areas under these variations did not exceed 0.64% (Table 5).

**Table 5:** Robustness assessment of the proposed HPLC method for the determination of TIR and SEM

Parameter	TIR				SEM			
	Mean rec.%± SD^a^	RSD %^b^	t_*r*_± SD^c^	AUC^d^	Mean rec.%± SD^a^	RSD %^b^	t_*r*_± SD^c^	AUC^d^
Flow rate, ml/min (± 0.2)	100.67 ± 1.23	0.61	1.93 ± 0.18	733.50	100.29 ± 1.22	0.61	1.38 ± 0.03	805.50
Detection wavelength, nm (± 2)	100.31 ± 1.03	0.52	1.68 ± 0.00	729.50	100.14 ± 0.46	0.23	1.42 ± 0.00	804.00
Mobile phase composition, % (± 2)	101.03 ± 1.31	0.65	1.68 ± 0.00	738.50	99.99 ± 0.75	0.37	1.42 ± 0.00	802.50

^a^Mean percentage recoveries of 200 µg/mL of each drug ± Standard deviation

^b^Percentage relative standard deviation

^c^Average of retention time ± standard deviation

^d^Area under the curve provided that the values of control AUC were 734.5 and 802 for TIR and SEM respectively

#### Stability of the solutions

Standard working solutions and sample solutions were tested for stability in the diluent (deionized water). No apparent degradation was found within 24 h at room temperature and in the refrigerator for a week and the retention times and peak areas of TIR and SEM remained constant (RSD% < 2%).

### Analysis of pharmaceutical dosage forms

The developed methods were applied for the estimation of TIR in Mounjaro^®^ and SEM in Ozempic^®^. Deionized water was used to dissolve the active ingredients from their respective dosage forms and for dilution to reach a specific concentration range. TIR and SEM eluted at their specific retention times with no interference from excipients as shown in Fig. 5. The peak purity of TIR and SEM indicated that there were no co-eluted inactive ingredients with them figure S2. The analysis results in Table 6a indicate high accuracy and precision of the developed method.

**Table 6:** Application of the proposed method for the quantitation of TIR in Mounjaro^®^and SEM in Ozempic^®^ and plasma

Drug	TIR			SEM		
a) Dosage form	Mounjaro^®^			Ozempic^®^		
Labeled concentration (µg/mL)	25	50	100	50	100	250
Found concentration (µg/mL)	24.77	50.75	100.78	49.74	101.70	248.40
%Recovery ± %RSD^a^	99.54 ± 0.66	100.75 ± 1.05	100.39 ± 0.55	99.74 ± 0.37	100.85 ± 1.19	99.678 ± 0.46
%Er^b^	-0.46	0.75	0.39	-0.26	0.85	-0.32
b) Plasma						
Labeled concentration (µg/mL)	100.00	200.00	300.00	100.00	200.00	300.00
Found concentration (µg/mL)	100.17	204.87	312.31	101.87	198.90	299.88
%Recovery ± %RSD^a^	100.17 ± 0.12	102.44 ± 1.70	104.10 ± 2.84	101.87 ± 1.31	99.45 ± 0.39	99.96 ± 0.03
%Er^b^	0.09	1.22	2.05	0.94	-0.26	-0.02
ANOVA						
^*^F	1.58			0.10		
P	0.27			0.95		

^a^Mean percentage recovery of three replicates ± percentage relative standard deviation

^b^Percentage relative error

^*^The critical F values were 4.07 and 4.06 for SEM and TIR respectively at p value = 0.05

No statistically significant differences were detected between the suggested and reported methods [16, 19] when one-way analysis of variance (ANOVA) was employed to assess the proposed methods for the analysis of TIR and SEM, Statistical comparison using ANOVA between the data obtained from the developed method and the reference method where no significant differences between the recoveries of the developed method and the reference ones (Table 6), which indicates the applicability of the developed methods for the estimation of TIR and SEM in injectable dosage forms with high precision, accuracy and selectivity.

### Analysis in plasma

**Fig. 6:**
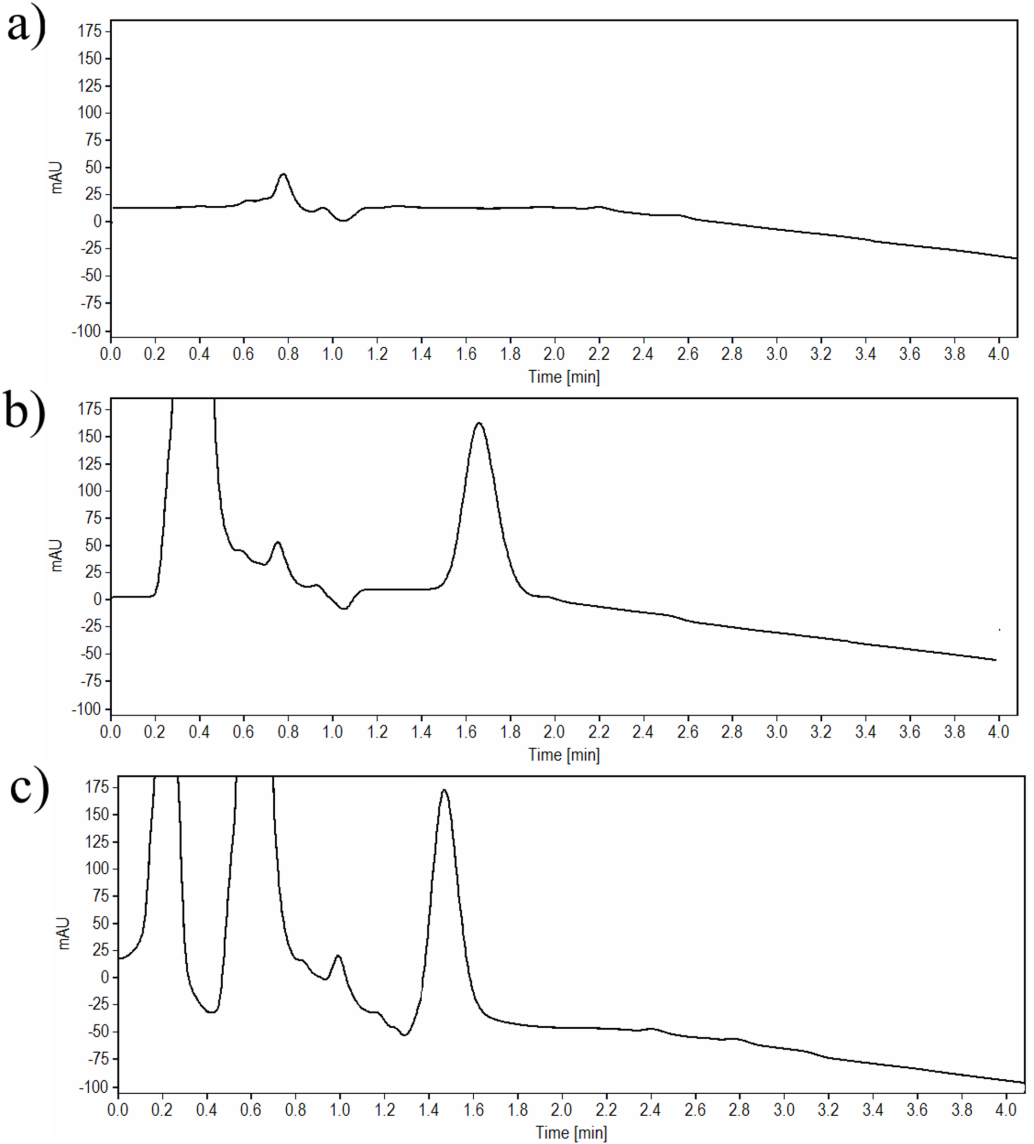
HPLC chromatograms of the proposed method showing **a** blank rat plasma, **b** 100 µL spiked rat plasma with 500 µg/mL TIR, **c** 100 µL spiked rat plasma with 500 µg/mL SEM measured at 220 nm

Considering the high sensitivity of the proposed HPLC method, which allows for accurate and precise determination of both drugs in bulk and dosage forms; plasma samples were spiked with TIR and SEM to clarify the ability of the proposed method in the quantification of the drugs in biological fluids for pharmacokinetic studies (Fig. 6). The samples were estimated as mentioned above and the results obtained are shown in Table 6b with mean percentage recovery ranging from 99.45% to 104.10% where the %RSD did not exceed 2.84%.

## Hexalateral sustainability assessment

### Greenness

The evaluation of the environmental impact of emerging analytical methods has increased in recent years to allow for unbiased comparison of different analytical techniques. Recently, many different evaluation metrics have been established including the GAPI, Analytical Eco-Scale, AGREE, MoGAPI, AGSA, and CaFRI.

#### Green analytical procedure index (GAPI)

GAPI is a systematic tool that evaluates the environmental performance of the analytical method. It is a visual system composed of five pictograms whose color varies according to the degree of greenness from red, yellow or green. It evaluates multiple steps in the analysis process as sample collection, preservation, storage and transportation. Moreover, the scale of analysis, safety and environmental impact of the used solvents and reagents are also considered [26]. One of the limitations of GAPI is that it is a qualitative tool and to overcome Complementary Green Analytical Procedure Index (Complex GAPI) software. Complex GAPI combines the advantages of GAPI and quantitative metrics as analytical Eco-Scale allowing for straightforward, quantitative and accurate assessment of analytical methodologies [27, 28].

#### Modified green analytical procedure index (MoGAPI)

Another recently developed greenness assessment tool is the Modified Green Analytical Procedure Index (MoGAPI) which is a modification of the original GAPI metric that uses a visual pictogram to assess the greenness of the analytical techniques. MoGAPI serves as both visual and quantitative assessment tool as it provides a point-based system in the form of a percentage. It assesses the different steps in the procedure, such as sample collection, preparation procedures, reagents used, consumed energy, waste amount and recycling along with the safety of the analyst through studying the occupational hazard. The greenness of the method is considered excellent if the obtained score is more than 75%, acceptable if for scores between 50 and 74%, and inadequate in the case of scores falling below 50% [29, 30]. Table 7 demonstrates the obtained score upon evaluation of the proposed HPLC method on the MoGAPI scale with a score of 82% confirming the greenness of the method.


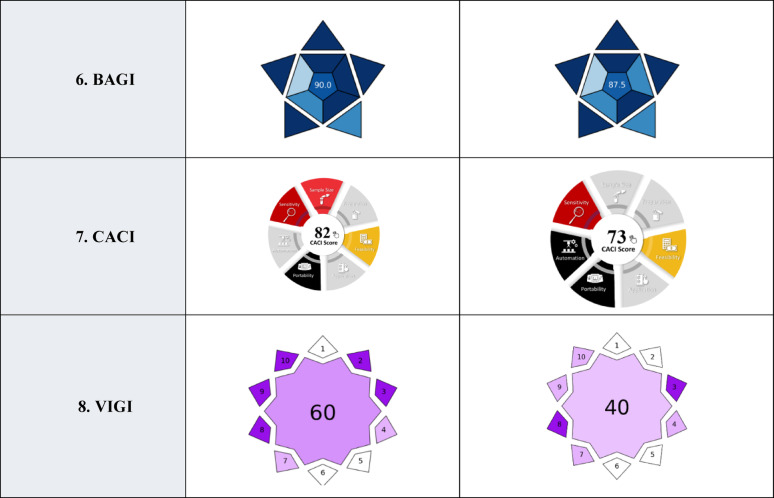

**Table 7:**
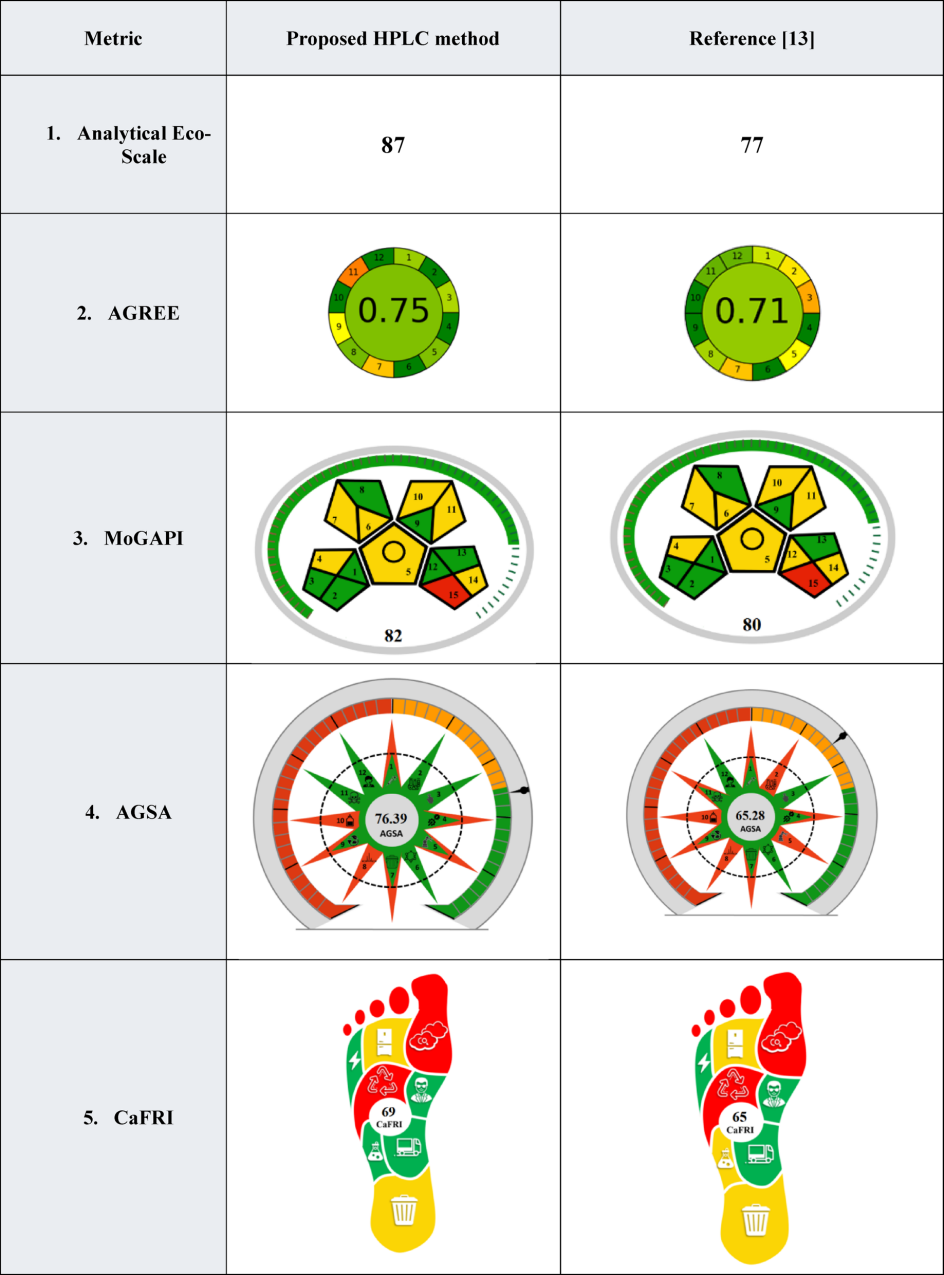
Sustainability assessment of the proposed method in bulk, dosage forms versus plasma using analytical Eco-Scale, AGREE, GAPI, MoGAPI, AGSA, CaFRI, BAGI, CACI, VIGI and STABLE metrics

*AGREE : Analytical Greenness Metric

*MoGAPI* : Modified Green Analytical Procedure Index

*AGSA* : Analytical Green Star Area

*CaFRI* : Carbon Footprint Reduction Index

BAGI: Blue Applicability Grade Index,

CACI : Click Analytical Chemistry Index ,

VIGI : Violet Innovation Grade Index

#### Analytical Eco-Scale (AES)

Analytical Eco-Scale (AES) is a quantitative tool used to assess the environmental impact of analytical methods by evaluating the method based on several criteria. These include waste generation, energy consumption, volume, safety of used reagents, method simplicity, time efficiency and operational factors. The base score is 100 and penalties are subtracted from 100 to obtain the method’s score [31].

Table 7 demonstrates the obtained score upon evaluation of the proposed HPLC method using the AES assessment system. In general, a score greater than 75 indicates a sustainable and environmentally friendly method. Table S1 lists the penalty points used in the calculation of the AES score. The proposed HPLC method obtained a score of 87 that proves its eco-friendliness (Table 7). 

#### Analytical Greenness Matric (AGREE)

AGREE is a greenness metric based on the twelve principles of Green Analytical Chemistry (GAC). It is based on a clock-shaped diagram composed of 12 sections where each one is corresponding to one principle of GAC principles. The color of each section is dependent on the compliance of the method with GAC principles and varies from red, yellow or green. AGREE is quantitative greenness assessment tool as it represents the overall score of the method at the center of the graph ranging from 0 to 1 [32]. A method is considered “sufficiently green” if it achieves a score ≥ 0.5 on the AGREE scale. Table 7) demonstrates the obtained diagram upon evaluation of the proposed HPLC method using the AGREE metric where the described method was found to be sufficiently green with a score of 0.75.

#### Analytical green star area (AGSA)

In view of the limitations existing in various greenness tools including NEMI, GAPI or AGREE, such as subjectivity in evaluation or lack of an overall score, the Analytical Green Star Area (AGSA) was designed. This assessment tool combines a visual representation as a star-shaped pictogram and a numerical score, making the comparison between different analytical techniques easier. AGSA is based on the principles of GAC and covers them in the evaluation in a systematic approach, and each principle is weighed in the final score including the toxicity of used solvents and reagents, minimization of the waste, consumed energy, safety and other factors. The star-shaped chart visually represents the degree of compliance with each principle, helping analysts quickly identify strengths and weaknesses of an investigated method [33]. Table 7 demonstrates the obtained diagram upon evaluation of the proposed HPLC method using the AGSA assessment tool where the proposed HPLC method has scored an acceptable greenness score of 76.39 on the AGSA scale.

#### Carbon footprint reduction index (CaFRI)

Carbon Footprint Reduction Index (CaFRI) is a recently designed tool used to evaluate the environmental impact of analytical methodologies, focusing on the reduction of carbon footprint. Unlike other greenness assessment metrics, which focus on the toxicity and safety of the solvents and reagents used, CaFRI prioritizes the reduction of carbon footprint. CaFRI emphasizes quantifying the greenhouse gases that are emitted directly or indirectly through energy consumption, recycling practices followed in the labs, and the requirements of the sample for storage, transportation, and analyst involvement. The total score ranges from 0 to 100, enabling researchers to easily compare between different analytical procedures. Nonetheless, CaFRI suffers from some drawbacks, including reliance on estimated data for energy use and throughput, subjectivity in scoring, and variability due to country-specific CO₂ emission factors. It simplifies complex life cycle impacts, and results may differ between laboratories with similar methods [34]. Table 7 demonstrates the obtained diagram upon evaluation of the proposed HPLC method using the CaFRI. The score of the proposed method was compared with a previously reported method [13] and the proposed method was found to be of lower Carbon footprint impact as it has higher CaFRI score 69 versus 65 for the reference method.

### Whiteness

**Fig. 7:**
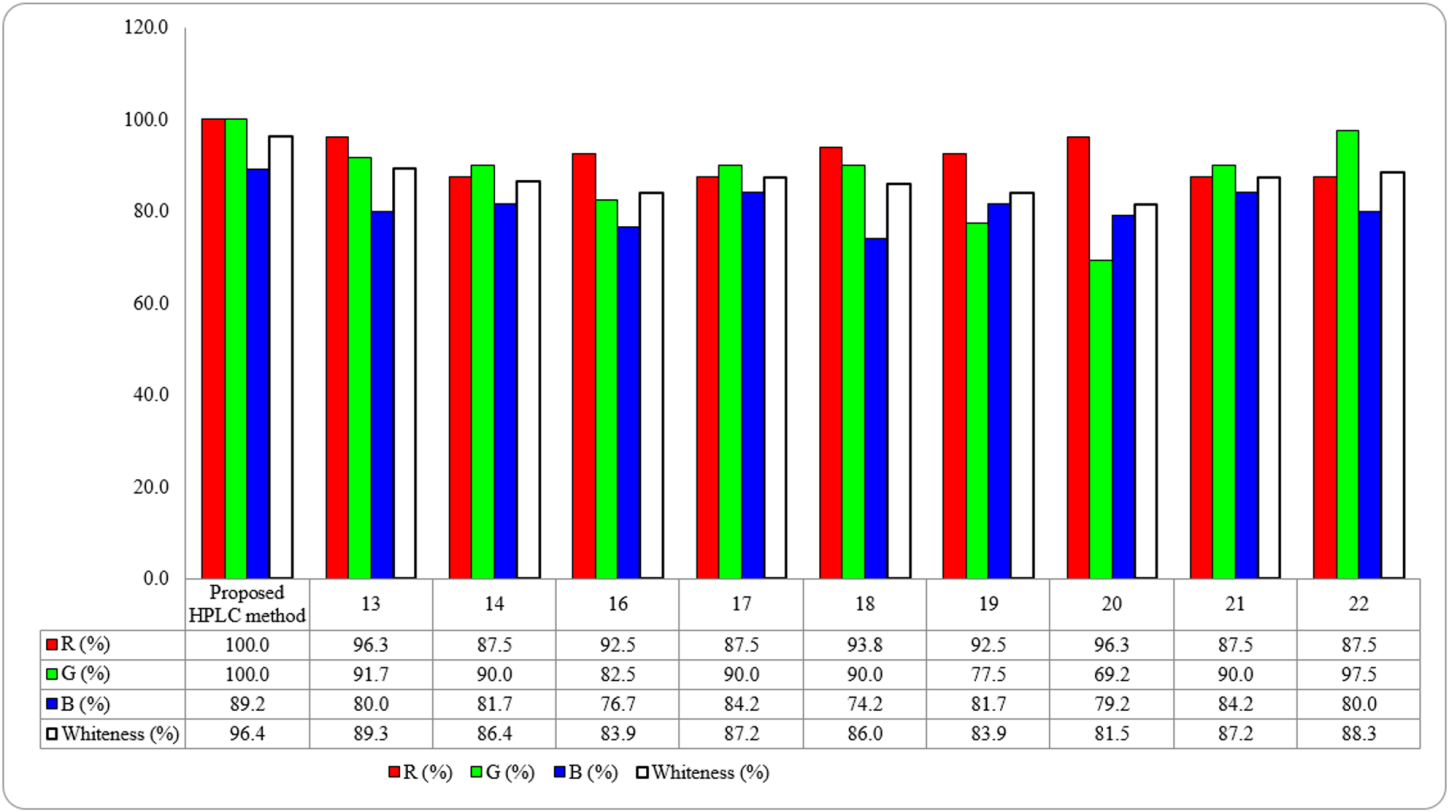
Whiteness assessment using RGB algorithm of the proposed HPLC method against reported methods for TIR and SEM deter

Recently, the concept of whiteness in analytical chemistry (WAC) was expanded from the concepts of GAC, which is a comprehensive approach that relies on three dimensions. The red dimension evaluates the analytical performance of the developed method by comparing the validation parameters of the analytical techniques as LOD, LOQ, linearity, accuracy, and precision. The higher the red score, the higher the validity of the developed method. The green dimension assesses the environmental sustainability by evaluating the type and amount of reagent used, solvents, energy and waste. The higher the green score, the higher the environmental compliance of the method. The applicability of the method is evaluated via the blue score which emphasizes the instrument availability, speed, cost and operator requirements. A high score indicates the reproducibility and accessibility of the method. This model is known as RGB model as it is a combination of the three basic colors; red, green, and blue produces a white color. The closer the obtained value to 100. the closer is the method is to ideal whiteness [35]. Figure 7 demonstrates the overall efficiency of the suggested method compared to other reported methods. Table S2 displays the obtained results upon evaluation of the whiteness of the proposed method in comparison with other reported methods in the literature. The superiority of the developed method compared to other reported methods is confirmed with its whiteness score approaching 97% against values ranging from 81.5 to 89.3% for the reported methods. The results of comparison are illustrated in table S2 [36–38].

### Blueness

Blueness is a computational and visual approach that emphasizes practicality, applicability, and compliance with environmental and operational goals of the developed analytical methods using a simplified desktop program called BAGI. This metric tool considers multiple parameters, including sample pretreatment, the amount of sample needed to complete the analysis, the speed of analysis, the degree of automation, the types of reagents utilized, and other factors. The results are presented as an asteroid pictogram with a distinct blue shade, the darker the blue, the greater is the compliance of the method with the set of criteria. A minimum score of 60 is recommended to ensure the practicality of the analytical technique [39]. Table 7 demonstrates the obtained diagram upon evaluation of the proposed HPLC method using the BAGI tool where the applicability of the suggested method is confirmed with a score of 90.

### Click analytical chemistry index (CACI)

A novel concept known as click analytical chemistry emphasizes usability and practicality of an analytical method without compromising its performance. CACI is a metric used to calculate the score of each analytical method and facilitates the comparison between different analytical techniques. CACI is considered complementary to BAGI to assess the practicality of the analytical techniques. Its score is dependent on evaluating eight parameters, such as the sample size, requirements to prepare the samples, degree of automation, instrumental portability, feasibility of the used chemicals and instruments, applicability, sensitivity of the method and the consumed analysis time. It uses a color-coded pictogram to represent the analytical method performance visually in a user-friendly pattern [40]. Table 7 demonstrates the obtained diagram upon evaluation of the proposed HPLC method using the CACI tool. The high practicality of the proposed HPLC method is further confirmed with CACI score of 82.

### Violet innovation grade index (VIGI)

VIGI is a novel survey-based evolution tool that assesses the degree of innovation of the analytical techniques. This evolution is based on ten innovation attributes including the use of advanced techniques used, sample preparation and preconcentration which reduces solvent consumption and offer enhanced sensitivity, In addition the criteria includes the use of advanced software and algorithms in data processing to reduce the human error, adherence to the principles of white analytical chemistry which combine greenness and blueness and the use of available tools as AGREE, GAPI and BAGI to ensure that the developed method is environmentally benign. The VIGI tool is also stresses on the alignment of the method with local and international standards, use of novel materials, technologies and robotics, and the interdisciplinarity of the method. Each attribute is scored 0, 5 or 10 from low innovation to high innovation and the sum of all attributes gives a score out of 100, a score ≥ 50 is considered innovative. The visual representation is a violet star-shaped decagon with varying violet shades [41].

Table 7 demonstrates the obtained diagram upon evaluation of the proposed HPLC method using the VIGI tool. With a VIGI score of 60, this work uncovers a first-time multidisciplinary approach for the analysis of TIR and SEM in their raw materials, dosage forms, plasma as well as stability indicating aspects that provides the method with unequivocal superiority among the reported methods which corresponds to principle [10]. This is further supported by the obtained nanogram level values of the LOD and LOD achieved using the proposed method which corresponds to principle [9].

### Stability toolkit for the appraisal of Bio/Pharmaceuticals’ level of endurance (STABLE)

STABLE is designed to quantify and standardize the stability of active pharmaceutical ingredients under different degradation conditions. Most guidelines define what to test in degradation studies such as the condition to test as acidic or basic degradation, oxidation, light and heat degradation but there is much flexibility in which those experiments are applied, raising inconstancies between different techniques. STABLE focuses on five stress conditions, acidic, basic, oxidative, thermal and photo degradation and provides a color-coded scoring system to evaluate the performance of the active pharmaceutical ingredient in each condition. The scoring in each condition depends on the concentration of the used reagent, exposure time, temperature of the experiment, or light intensity in case of photo degradation and the observed percentage of degradation. The drug is considered to have high stability if the percentage degradation is less than 10% under severe stress conditions and vice versa. STABLE fills the gap by providing a numerical and visual summary which helps in the comparison of the stability of different drugs transparently [42] and when the result quantified using STABLE, TIR and SEM where found to be of similar mixed stability with scores of 61 and 60 for TIR and SEM, respectively (Table 8).

**Table 8:**
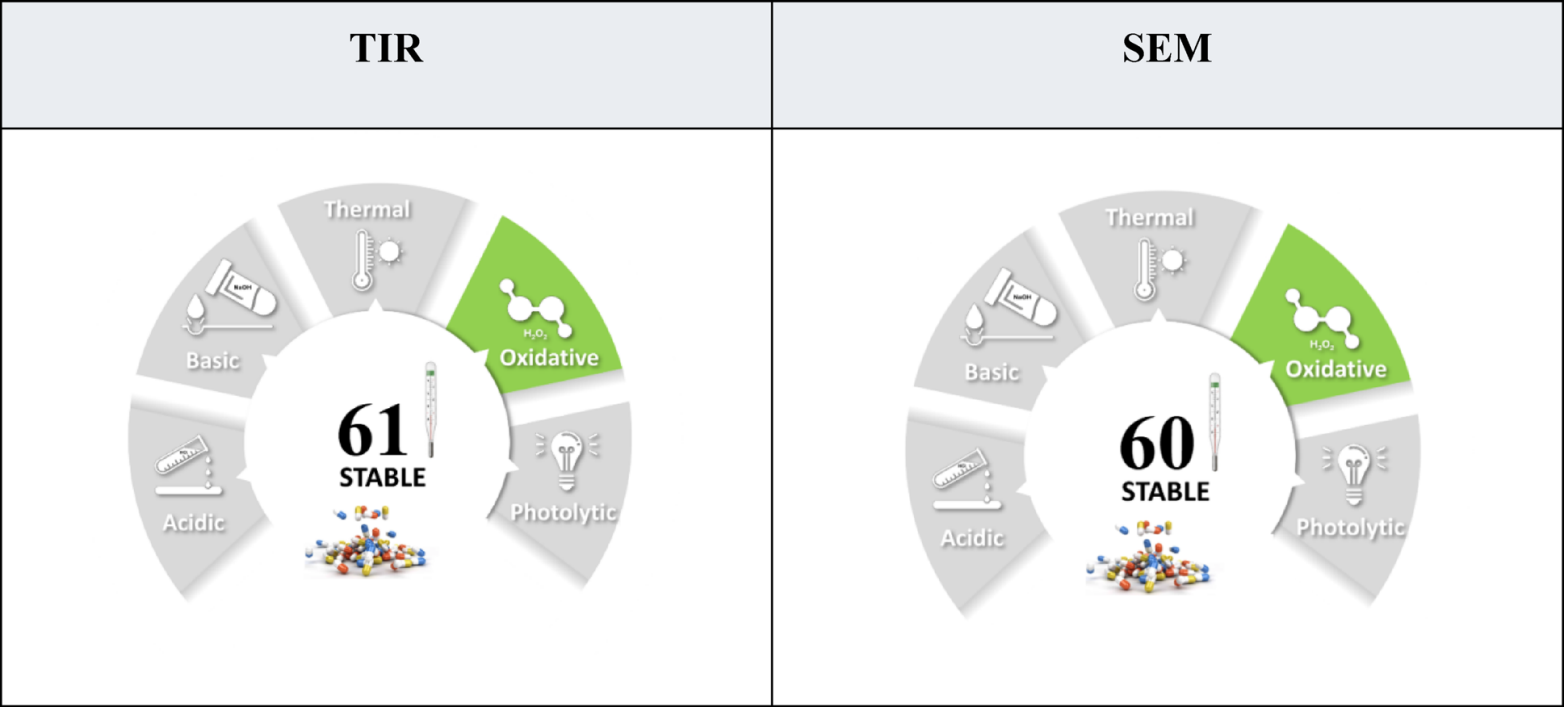
Stability evaluation of the studied drugs, TIR and SEM under the proposed degradation conditions using STABLE metric

## Conclusion

This work presents the first validated stability-indicating HPLC method with significant degradation results for the determination of TIR and SEM under hydrolytic (acid, base and neutral), oxidative, and photolytic stress conditions. TIR and SEM were found to be degraded in 0.1 M NaOH, 0.1 M HCl ,10% H_2_O_2_, thermal and photolytic conditions. The proposed HPLC method enables the separation of all the degradation products from the studied drugs confirming the selectivity and specificity of the method. The method was successfully validated according to the ICH guidelines and was effectively employed to determine each drug in bulk, pharmaceutical dosage forms, and spiked rat plasma. The proposed method has a short retention time for both TIR and SEM which may cause overlapping of the peaks with poorly retained substances due to poor interaction with stationary phase. A comprehensive hexahedral sustainability assessment of the proposed method was conducted using greenness, whiteness, blueness, innovation, carbon footprint and stability metrics including AGREE, MoGAPI, Analytical Eco-scale, AGSA, CaFRI, Whiteness using RGB algorithm, BAGI, CACI, VIGI tools and STABLE. The obtained scores confirmed that the proposed method is sustainable, environmentally benign and superior to previously reported methods in terms of novelty, sensitivity, and applicability. This renders the method an invaluable broad spectrum analytical tool for laboratory quality control purposes as well as in pharmacokinetic investigations of TIR and SEM.

## Supplementary Information

Below is the link to the electronic supplementary material.

Supplementary Material 1.

## Data Availability

The datasets generated or analysed during this study are available from the corresponding author on reasonable request.

## Abbreviations

TIR: Tirzepatide
SEM: Semaglutide
AGREE: Analytical greenness metric
GAPI: Green analytical procedure index
MoGAPI: Modified green analytical procedure index
AGSA: Analytical eco-scale, analytical green star area
CaFRI: Carbon footprint reduction index
BAGI: Blue applicability grade index
CACI: Click analytical chemistry index
VIGI: Violet innovation grade index
STABLE: Stability toolkit for the appraisal of bio/pharmaceuticals’ level of endurance
GLP-1: Glucagon-like peptide-1
GIP: Glucose-dependent insulinotropic polypeptide
HPLC-DAD: High-performance liquid chromatographic method with diode array detection
LC-MS/MS: Liquid chromatography with tandem mass detection
UPLC: Ultra-performance liquid chromatography
ACN: Acetonitrile
SIAM: Stability-indicating assay method
S_a_: Standard deviations of the intercept
S_b_: Slope
S_y/x_: Standard deviations of residuals
LOD: Limit of detection
LOQ: Limit of quantification
RSD%: Percentage relative standard deviation
Er%: Percentage relative error
GAC: Green analytical chemistry
t_R_: Retention time
K`: Capacity factor
T: Tailing Factor
N: Theoretical plates
