# Supplementary material for: A multimodal HPLC stability indicating approach for the estimation of Semaglutide and Tirzepatide in bulk, pharmaceutical dosage forms, and rat plasma: a six-edged sustainability appraisal
Source: BMC Chem. 2026 Jan 27;20(1):31. doi: 10.1186/s13065-025-01716-7 (PMC12918740; doi:10.1186/s13065-025-01716-7)

**Supplementary Material**

**Table S1: Penalty points for the determination of TIR and SEM using the proposed HPLC method.**

| **Evaluation Parameter** | **Proposed HPLC Method** | **Reference [13]** |
| --- | --- | --- |
| **ACN** | 6 | 4 |
| **Ethanol** | - | 16 |
| **Formic acid** | 3 | - |
| **Distilled water** | 0 | 0 |
| **Energy** | 1 | 0 |
| **Occupational Hazard** | 0 | 0 |
| **Waste** | 3 | 3 |
| **Total penalty points** | 13 | 23 |
| **Analytical Eco-Scale Total Score** | 87 | 77 |

**Table S2: WAC Approach for Assessment of Validation, Greenness , Practicality of the proposed HPLC method compared with the reported method s from literature**.

| **Principles of WAC** | **Proposed HPLC method** | **[13]** | **[14]** | **[16]** | **[17]** | **[18]** | **[19]** | **[20]** | **[21]** | **[22]** |
| --- | --- | --- | --- | --- | --- | --- | --- | --- | --- | --- |
| **R1—linearity and validation** | 100 | 90 | 70 | 80 | 70 | 80 | 80 | 90 | 70 | 70 |
| **R2—specificity and selectivity** | 100 | 95 | 80 | 90 | 80 | 95 | 90 | 95 | 80 | 80 |
| **R3—accuracy** | 100 | 100 | 100 | 100 | 100 | 100 | 100 | 100 | 100 | 100 |
| **R4— precision** | 100 | 100 | 100 | 100 | 100 | 100 | 100 | 100 | 100 | 100 |
| **R model score** | 100 | 96.25 | 87.5 | 92.5 | 87.5 | 93.75 | 92.5 | 96.25 | 87.5 | 87.5 |
| **G1— Toxicity of reagents (impact and biodegradation)** | 100 | 100 | 100 | 80 | 100 | 100 | 60 | 60 | 100 | 100 |
| **G2: Amount of reagents and waste** | 100 | 100 | 80 | 70 | 80 | 80 | 70 | 80 | 80 | 90 |
| **G3—power consumption** | 100 | 100 | 80 | 80 | 80 | 80 | 80 | 70 | 80 | 100 |
| **G4: Direct impacts (safety, use of animals and GMOs)** | 100 | 100 | 100 | 100 | 100 | 100 | 100 | 100 | 100 | 100 |
| **G model score** | 100 | 91.7 | 90 | 82.5 | 90 | 90 | 77.5 | 69.2 | 90 | 97.5 |
| **B1: Cost-efficiency** | 90 | 100 | 90 | 90 | 90 | 70 | 90 | 90 | 90 | 100 |
| **B2: Time-efficiency** | 100 | 70 | 70 | 50 | 80 | 70 | 70 | 60 | 80 | 70 |
| **B3: Requirements** | 100 | 90 | 100 | 100 | 100 | 90 | 100 | 100 | 100 | 90 |
| **B4: Operational simplicity** | 66.7 | 60 | 66.7 | 66.7 | 66.7 | 66.7 | 66.7 | 66.7 | 66.7 | 60 |
| **B model score** | 89.2 | 80.0 | 81.7 | 76.7 | 84.2 | 74.2 | 81.7 | 79.2 | 84.2 | 80.0 |
| **WAC score (Average of RGB scores)** | 96.4 | 89.3 | 86.4 | 83.9 | 87.2 | 86.0 | 83.9 | 81.5 | 87.2 | 88.3 |
| **WAC status** | Excellent white | Excellent white | Excellent white | Excellent white | Excellent white | Excellent white | Excellent white | Excellent white | Excellent white | Excellent white |

**Figure S1. Peak purity plots (a-e) of 100 μg/ml solution of TIR after exposure to a) acid degradation, b) basic degradation, c) oxidative degradation, d) photolytic degradation, and e) thermal wet heat degradation. Peak purity plots (f-j) of 100 μg/ml solution of**[**SEM**](https://www.sciencedirect.com/topics/pharmacology-toxicology-and-pharmaceutical-science/hydrochlorothiazide)**after exposure to f) acid degradation, g)**[**basic**](https://www.sciencedirect.com/topics/pharmacology-toxicology-and-pharmaceutical-science/base)**degradation, h)**[**oxidative degradation**](https://www.sciencedirect.com/topics/chemistry/oxidative-degradation)**, i) photolytic degradation, and j) thermal wet heat degradation.**


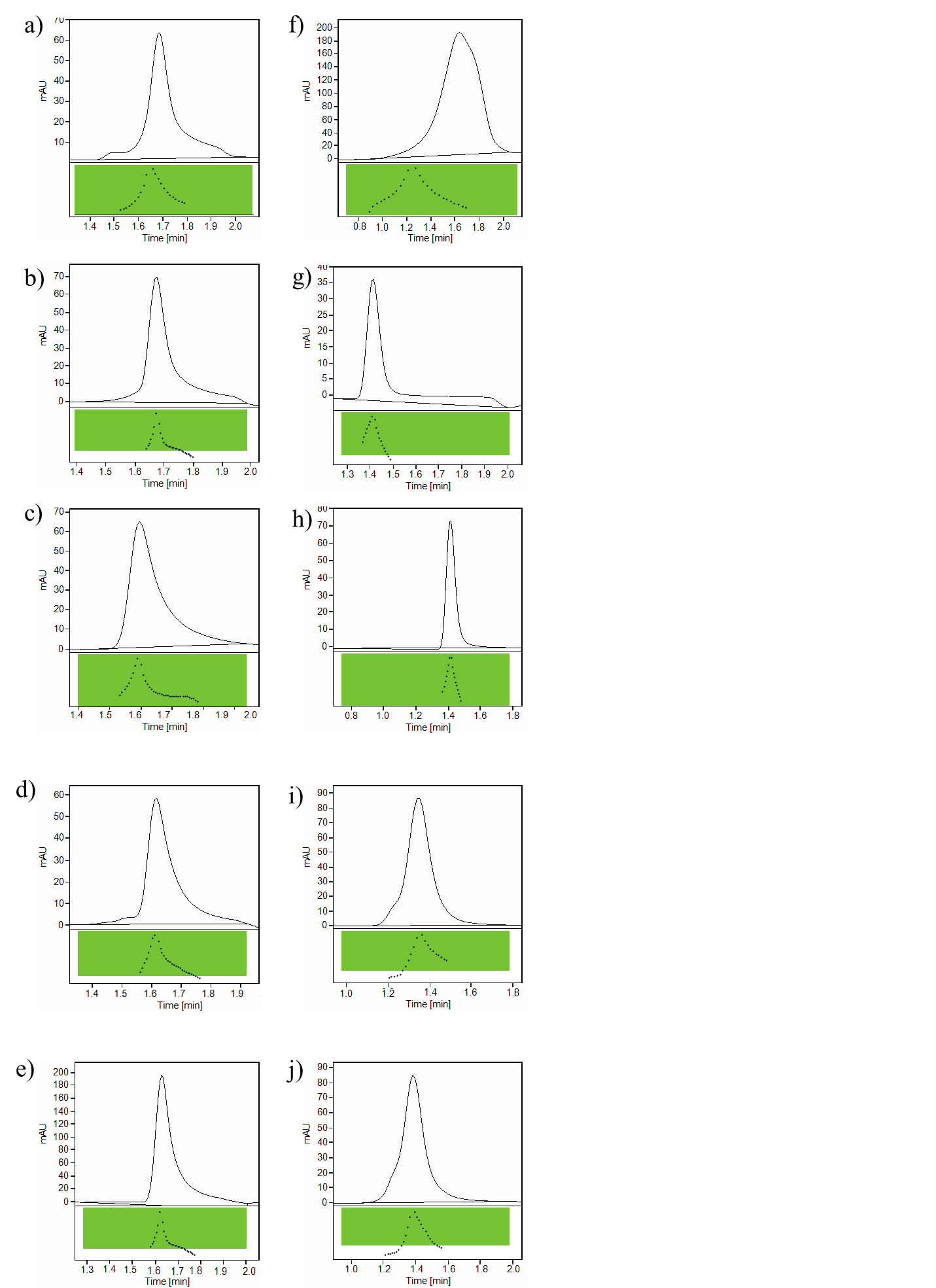


**Figure S2. Peak purity plots (a) of 100 μg/mL solution of Mounjaro^®^ and b) 100 μg/mL solution of Ozempic^®^.**


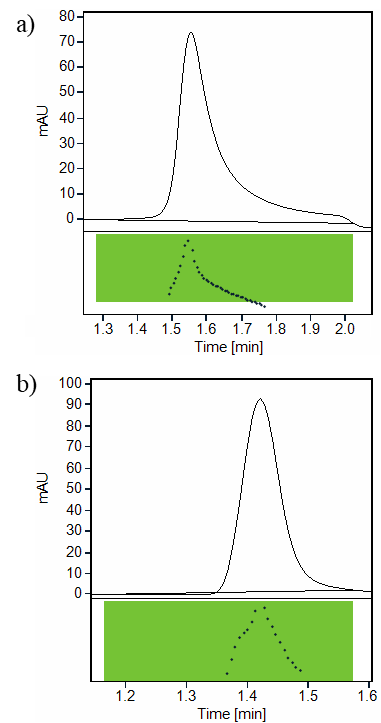

Supplement: Supplementary file 1 — Supplementary Material 1. [file 13065_2025_1716_MOESM1_ESM.docx]
